# Flame-Oxidized Stainless-Steel Anode as a Probe in Bioelectrochemical System-Based Biosensors to Monitor the Biochemical Oxygen Demand of Wastewater

**DOI:** 10.3390/s18020607

**Published:** 2018-02-16

**Authors:** Qiaochu Liang, Takahiro Yamashita, Ryoko Yamamoto-Ikemoto, Hiroshi Yokoyama

**Affiliations:** 1 Graduate School of Nature Science & Technology, Kanazawa University, Kakumamachi Kanazawa, Ishikawa 920-1192, Japan; qiaochu@stu.kanazawa-u.ac.jp (Q.L.); rikemoto@se.kanazawa-u.ac.jp (R.Y.-I.); 2 Division of Animal Environment and Waste Management Research, Institute of Livestock and Grassland Science, National Agriculture and Food Research Organization (NARO), 2 Ikenodai, Tsukuba 305-0901, Japan; yamatktk@affrc.go.jp

**Keywords:** biochemical oxygen demand, bioelectrochemical system, biosensor, flame oxidation, livestock wastewater, metal anode, stainless steel

## Abstract

Biochemical oxygen demand (BOD) is a widely used index of water quality in wastewater treatment; however, conventional measurement methods are time-consuming. In this study, we analyzed a novel flame-oxidized stainless steel anode (FO-SSA) for use as the probe of bioelectrochemical system (BES)-based biosensors to monitor the BOD of treated swine wastewater. A thinner biofilm formed on the FO-SSA compared with that on a common carbon-cloth anode (CCA). The FO-SSA was superior to the CCA in terms of rapid sensing; the response time of the FO-SSA to obtain the value of *R*^2^ > 0.8 was 1 h, whereas the CCA required 4 h. These results indicate that the FO-SSA offers better performance than traditional CCAs in BES biosensors and can be used to improve biomonitoring of wastewater.

## 1. Introduction

Biochemical oxygen demand (BOD) is a widely used index for measuring the amount of biodegradable organic matter in wastewater. Since the conventional BOD measurement method is time-consuming (5 days), more rapid techniques are required to support advances in wastewater-treatment processes. BOD biosensors based on microbial fuel cells (MFCs) have emerged as an alternative to the conventional method [1]. In MFCs, exoelectrogenic bacteria adhere to the anode surface and transfer electrons from organic matter to the anode under anaerobic conditions. Kim et al. [2] reported on a mediator-free MFC-based BOD biosensor for the first time. Di Lorenzo et al. [3] reported on a single-chamber MFC-based biosensor that measured chemical oxygen demand values up to 350 ppm with a coefficient of determination (*R*^2^) > 0.96 using artificial wastewater. Recently, a self-powered MFC-based biosensor was developed for online BOD monitoring [4]. At present, there is only one MFC-based biosensor available for sale by a Korean company (KORBI, Seoul, Korea). Current generation in MFC-based biosensors depends not only on the anode reaction, but also on the cathode reaction, and changes in cathode conditions can potentially affect current generation in a BOD-independent manner. To circumvent the cathode dependency, we recently developed a novel bioelectrochemical system (BES)-based BOD biosensor equipped with a potentiostat [5]. The anodic potential of the biosensor is kept constant by the potentiostat; therefore, the current generation is expected to depend on only the anode reaction based on the potential control. 

Usually, carbonaceous electrodes, such as carbon cloth, carbon felt, and carbon fiber, are used as the anodes in MFCs, since they have large effective surface areas and high biocompatibility with microbes. A new method that oxidizes the surface of a stainless steel anode (SSA) using fire reportedly improves the current output in BESs [6] and power production in MFCs [7]. The maximum power density using a flame-oxidized (FO)-SSA in MFCs was 24% higher than that using a carbon-cloth anode (CCA) [7]. Flame oxidation of stainless steel leads to the formation of Fe oxide nanoparticles on the surface, which have been suggested to attract exoelectrogenic bacteria that prefer Fe oxides, such as *Geobacter* spp., onto the surface in MFCs [7]. *Geobacter* spp. are representative exoelectrogenic bacteria with Fe oxide-reducing activity [8].

The performance of FO-SSAs has been characterized in MFCs, but not in BOD biosensors. Therefore, in the present study, we analyzed the utility of an FO-SSA as the probe of a BES-based biosensor for BOD measurements. Most studies on BOD biosensors have used artificial wastewater. However, evaluations using real wastewater are ultimately necessary for the practical application of biosensors, since the structure of the anode biofilm is dependent on the medium supplied. Therefore, we fed real treated swine wastewater into the BES-biosensor, and the performance of the FO-SSA was compared with that of a typical CCA.

## 2. Materials and Methods

### 2.1. Biosensor Construction

**Figure 1:**
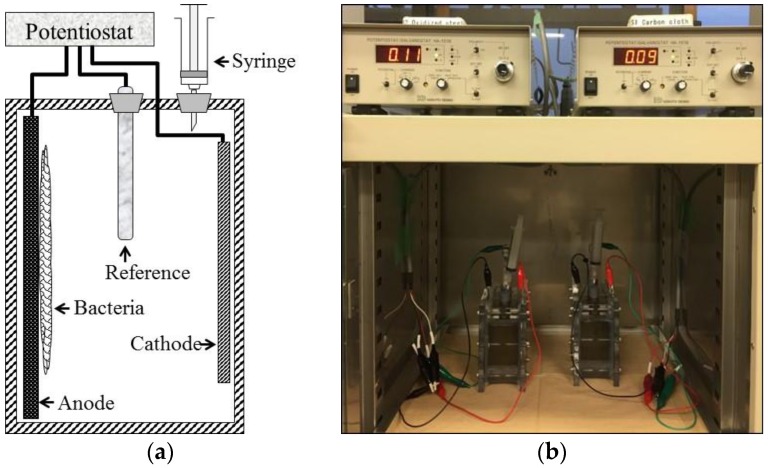
(**a**) Schematic representation and (**b**) photograph of the BES-based BOD biosensor used in this study.

The biosensor was rectangular (dimensions: 80 mm length × 50 mm width × 70 mm height; Figure 1). A mesh-shaped FO-SSA (70 mm × 80 mm × 0.2 mm) or CCA (70 mm × 80 mm × 0.2 mm) was placed inside the reactor. The FO-SSA was prepared by flame-oxidizing stainless-steel mesh (#60 mesh, SUS304) for 10 min, as described previously [7]. A plate-shaped stainless-steel cathode (SUS304, 50 mm × 80 mm × 0.2 mm) was placed opposite the anode in the reactor. An Ag/AgCl double-junction type reference electrode was inserted into the reactor, and the three electrodes were connected to a potentiostat (HA-151B; Hokuto Denko, Tokyo, Japan). A syringe was inserted in the top of the reactor to prevent increases in the inner pressure due to CO_2_ production.

### 2.2. Biosensor Operation and Analysis

The anode potential was set to −0.2 V (vs. Ag/AgCl). The reactor was inoculated with active sludge collected from an animal wastewater treatment plant at the Institute of Livestock and Grassland Science (Tsukuba, Japan) as seed sludge. During an acclimation period of 3 weeks, raw swine wastewater (>500 mg BOD/L) was fed into the reactors, and medium exchange was conducted three times per week. Treated swine wastewater, purified using a conventional activated sludge process, was settled for 1 h, and the supernatant (8–90 mg BOD/L) was subsequently supplied to the biosensors. The biosensors were operated at 30 °C under repeated-batch culture mode. The whole volume of the reactor content was manually replaced with fresh medium using a syringe for each batch culture. Current generation was recorded every 15 min with a data logger. BOD was measured using a conventional respirometric method (BOD_5_) at 20 °C using an apparatus equipped with a pressure sensor. To quantify the biofilm amount, the anodes after culture were dried at 110 °C for 24 h and then cooled in a desiccator for 24 h. The mass of the attached biofilm was estimated by subtracting the weight of the anodes before use from the weight after use.

## 3. Results and Discussion

### 3.1. BOD Monitoring

**Figure 2:**
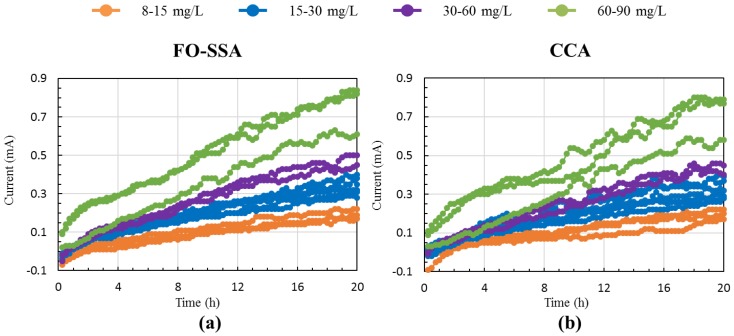
Current generation profiles of (**a**) FO-SSA and (**b**) CCA fed with the indicated concentrations of BOD_5_.

**Figure 3:**
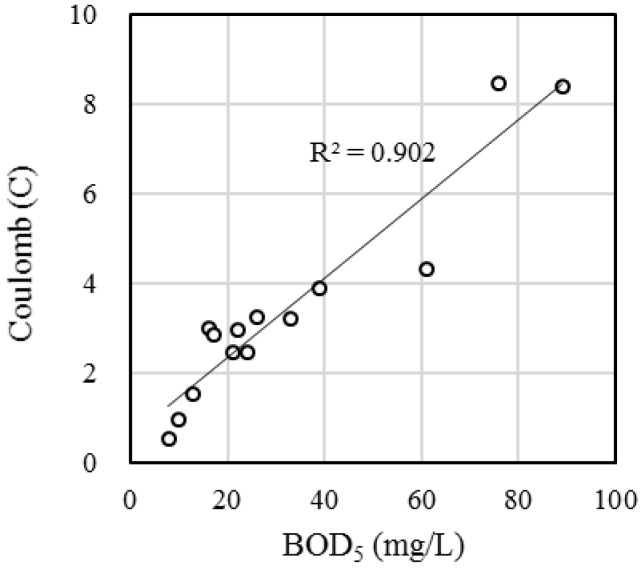
Correlation between coulomb and BOD_5_ at a response time of 8 h in the FO-SSA-equipped biosensor.

Figure 1 shows the configuration of the BES-based BOD biosensor. To analyze current response, various BOD_5_ concentrations were supplied to biosensors equipped with an FO-SSA or a CCA. The current intensity of both biosensors increased as the concentration of BOD_5_ increased (Figure 2). The time course of current generation was similar between the biosensors; in both, the current increased linearly and did not reach a plateau within 20 h. The *R*^2^ values of the correlation between coulombs (current × time) with BOD_5_ in both biosensors (*n* = 14) increased with increasing response time and reached > 0.9 (Table 1). The FO-SSA showed higher *R*^2^ values than CCA at all response times. The response time to obtain *R*^2^ > 0.8 was 1 h for the FO-SSA, whereas the CCA required 4 h to reach *R*^2^ > 0.8. In addition, *R*^2^ > 0.9 was achieved within a shorter time (8 h) with the FO-SSA than the CCA (12 h). Figure 3 presents the correlation between BOD_5_ and coulombs at a response time of 8 h using the FO-SSA. The FO-SSA showed a linear correlation within the tested range of 8–90 mg BOD_5_/L (*R*^2^ > 0.9). These results clearly show that FO-SSA offers superior performance to the CCA in terms of the rapid detection of BOD in wastewater.

### 3.2. Efficacy of the FO-SSA as a BES-Based BOD Biosensor Probe

**Figure 4:**
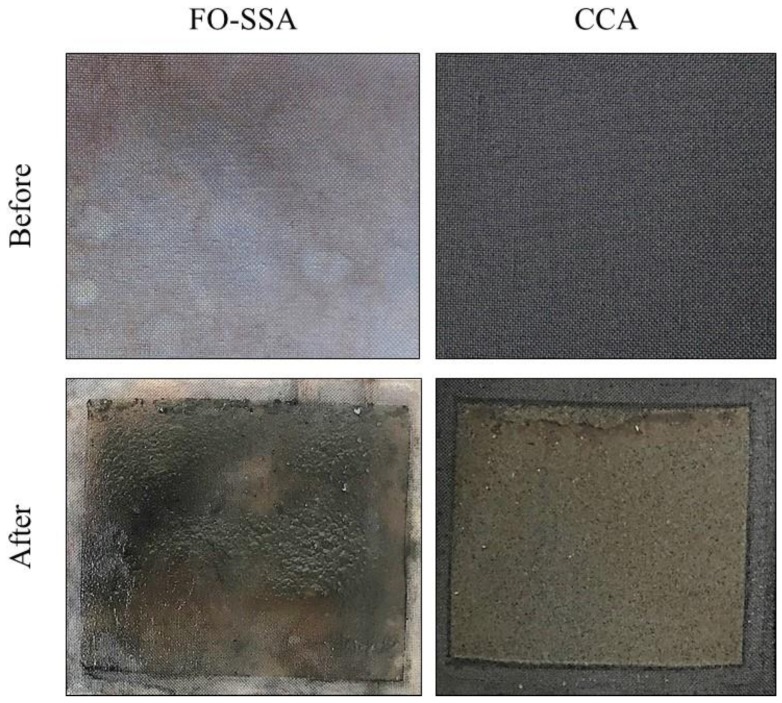
Images of the FO-SSA and CCA before and after culturing, showing biofilm attachment.

Metal-based anodes are rarely used in BESs, since bacteria adhere poorly to metal electrodes compared with carbon electrodes, and some metals are toxic to microbes. Nevertheless, we found that the FO-SSA enabled rapid BOD sensing. The biofilm on the FO-SSA appeared to be thinner than that on the CCA (Figure 4). To confirm this observation, the dry weight of the biofilms on the FO-SSA and CCA were determined. The biofilm on the FO-SSA (0.5 mg/cm^2^ projected surface area) was less than one-third of that on the CCA (1.8 mg/cm^2^ projected surface area). Owing to the thinness of the biofilm, substrates for current production in the wastewater might penetrate the biofilm on the FO-SSA more quickly than the CCA, resulting in a faster response of the FO-SSA-equipped biosensor to changes in BOD concentration. The structure of biofilms on BES anodes is dependent on the medium used. For example, when minimal medium with acetate for exoelectrogenic bacteria was supplied to BESs, a thin biofilm dominated by *Geobacter* spp. frequently formed on the anode [9]. In another case using real wastewater, a thick biofilm with a two-layer structure formed on the anode of BESs [5]. Exoelectrogenic bacteria are present inside the biofilm, and non-exoelectrogenic bacteria decomposing organic matter into smaller molecules are present outside the biofilm. Generally, the high biocompatibility of carbon electrodes, which leads to the abundant and rapid attachment of bacteria, is an advantageous feature in BESs. However, in the case of BOD biosensors for real wastewater, carbon electrodes might result in an overly thick biofilm layer, resulting in inaccurate and delayed sensing. We speculate that the anode biofilm thickness might be a critical parameter for the rapid sensing of BOD in real wastewater. Further studies are required to verify this hypothesis.

### 3.3. Potential Applications of FO-SSA-Equipped BOD Biosensors

Stainless steel is highly conductive compared with standard carbonaceous electrodes and is a low-cost material with good chemical and mechanical strength. FO-SSA preparation is a relatively simple process, which requires the short application of flame to stainless steel mesh. In this study, a BES-based biosensor was equipped with an FO-SSA in a manner that enabled the anode potential to be kept constant for accurate sensing. Water pollution is a major problem that damages water resources, such as rivers, ponds, and ground water, and various practical applications of the biosensor presented herein are expected. For example, the biosensor could be placed in the final sedimentation tank of wastewater treatment plants to prevent discharge of inadequately treated wastewater with high BOD concentrations into the surrounding aquatic environment linking an alarm to the biosensor.

## Figures and Tables

**Table 1:** *R*^2^ values of the correlation between coulomb and BOD_5_ at the indicated response times for biosensors equipped with an FO-SSA or a CCA (*n* = 14).

Response Time (h)	FO-SSA	CCA
1	0.805	0.696
2	0.823	0.752
3	0.845	0.789
4	0.860	0.803
5	0.874	0.820
6	0.884	0.836
7	0.894	0.851
8	0.902	0.867
10	0.918	0.895
12	0.924	0.912
16	0.937	0.930
20	0.941	0.939
